# Reproducible sex differences in personalised functional network topography in youth

**DOI:** 10.1192/bjp.2025.135

**Published:** 2025-06

**Authors:** Arielle S. Keller, Kevin Y. Sun, Ashley Francisco, Heather Robinson, Emily Beydler, Dani S. Bassett, Matthew Cieslak, Zaixu Cui, Christos Davatzikos, Yong Fan, Margaret Gardner, Rachel Kishton, Sara L. Kornfield, Bart Larsen, Hongming Li, Isabella Linder, Adam Pines, Laura Pritschet, Armin Raznahan, David R. Roalf, Jakob Seidlitz, Golia Shafiei, Russell T. Shinohara, Lauren K. White, Daniel H. Wolf, Aaron Alexander-Bloch, Theodore D. Satterthwaite, Sheila Shanmugan

**Affiliations:** Department of Psychological Sciences, University of Connecticut, Storrs, Connecticut, USA; Institute for the Brain and Cognitive Sciences, University of Connecticut, Storrs, Connecticut, USA; Lifespan Brain Institute (LiBI) of Penn Medicine and CHOP, University of Pennsylvania, Philadelphia, Pennsylvania, USA; The Penn Lifespan Informatics and Neuroimaging Center, University of Pennsylvania, Philadelphia, Pennsylvania, USA; Department of Psychiatry, University of Pennsylvania, Philadelphia, Pennsylvania, USA; Departments of Bioengineering, Electrical & Systems Engineering, Physics & Astronomy, and Neurology, University of Pennsylvania, Philadelphia, Pennsylvania, USA; Santa Fe Institute, Santa Fe, New Mexico, USA; Chinese Institute for Brain Research, Beijing, China; Department of Radiology, University of Pennsylvania, Philadelphia, Pennsylvania, USA; Center for Biomedical Image Computing and Analytics, University of Pennsylvania, Philadelphia, Pennsylvania, USA; Department of Family Medicine and Community Health, Penn Medicine, University of Pennsylvania, Philadelphia, Pennsylvania, USA; Penn Center for Women’s Behavioral Wellness, University of Pennsylvania, Philadelphia, Pennsylvania, USA; Masonic Institute for the Developing Brain, Institute of Child Development, University of Minnesota, Minneapolis, Minnesota, USA; Department of Pediatrics, University of Minnesota, Minneapolis, Minnesota, USA; Department of Psychiatry and Behavioral Sciences, Stanford University, Stanford, California, USA; Section on Developmental Neurogenomics, Human Genetics Branch, National Institute of Mental Health, Bethesda, Maryland, USA; Department of Child and Adolescent Psychiatry and Behavioral Science, Children’s Hospital of Philadelphia, Philadelphia, Pennsylvania, USA; Penn Statistics in Imaging and Visualization Center, Department of Biostatistics, Epidemiology, and Informatics, University of Pennsylvania, Philadelphia, Pennsylvania, USA; Department of Obstetrics and Gynecology, University of Pennsylvania, Philadelphia, Pennsylvania, USA

**Keywords:** Sex differences, brain networks, youth, development, precision brain mapping

## Abstract

**Background:**

A key step toward understanding psychiatric disorders that disproportionately impact female mental health is delineating the emergence of sex-specific patterns of brain organisation at the critical transition from childhood to adolescence. Prior work suggests that individual differences in the spatial organisation of functional brain networks across the cortex are associated with psychopathology and differ systematically by sex.

**Aims:**

We aimed to evaluate the impact of sex on the spatial organisation of person-specific functional brain networks.

**Method:**

We leveraged person-specific atlases of functional brain networks, defined using non-negative matrix factorisation, in a sample of *n* = 6437 youths from the Adolescent Brain Cognitive Development Study. Across independent discovery and replication samples, we used generalised additive models to uncover associations between sex and the spatial layout (topography) of personalised functional networks (PFNs). We also trained support vector machines to classify participants’ sex from multivariate patterns of PFN topography.

**Results:**

Sex differences in PFN topography were greatest in association networks including the frontoparietal, ventral attention and default mode networks. Machine learning models trained on participants’ PFNs were able to classify participant sex with high accuracy.

**Conclusions:**

Sex differences in PFN topography are robust, and replicate across large-scale samples of youth. These results suggest a potential contributor to the female-biased risk in depressive and anxiety disorders that emerge at the transition from childhood to adolescence.

Many psychiatric disorders show sex differences in prevalence, presentation and trajectory. For example, the lifetime prevalence of internalising disorders such as depression and anxiety is nearly twice as high in females,^1^ and developmental disorders such as attention-deficit hyperactivity disorder often present differently in males and females, leading to disparities in diagnosis and treatment. These sex differences tend to emerge during the transition from childhood to adolescence, a time when functional brain networks implicated in these disorders are refined.^2,3^ Previous research has begun to link sex differences in internalising disorders with sex differences in multimodal neuroimaging measures, including in studies of youth.^4–6^ Therefore, understanding and treating mental health conditions for all individuals, including those that are more prevalent in and differentially impact females, requires a clear understanding of sex differences in neurodevelopment.

Prior neuroimaging studies have revealed significant sex differences in functional networks supporting cognitive and emotional processes, including the frontoparietal^7,8^ and default mode^9^ networks. Dysfunction within these networks has been linked with psychiatric disorders, including anxiety and depression.^10–14^ Critically, these functional networks are highly person-specific in their spatial organisation across the cortex (‘functional topography’). Substantial individual differences in the size, shape and spatial location of brain regions comprising these networks emerge gradually during neurodevelopment, with evidence of sex-specific patterning^3,15^ associated with X-linked gene expression patterns.^15^ Innovations in precision brain mapping approaches have begun to chart the person-specific functional topography of personalised functional brain networks (PFNs),^16–18^ and have uncovered novel associations with internalising psychopathology^14,19,20^ and cognition.^3,21^

In a recent study of individuals across a broad age range (*n* = 693, 8–22 years old),^15^ we presented the first report of sex differences in PFN functional topography. Given the ongoing ‘reproducibility crisis’ in psychology and neuroscience wherein a large proportion of research findings fail to replicate in new data-sets,^22^ it is important to determine whether sex differences in functional topography are replicable across demographically diverse samples with a wider variety of magnetic resonance imaging (MRI) scanning locations and procedures. Moreover, it remains unclear whether these sex differences are consistently observed at the critical transition from childhood to adolescence when many psychiatric disorders first emerge, and whether these differences are associated with pubertal hormone levels. Here we examine sex differences in PFN topography in youth, using non-linear modelling and machine learning in data from the Adolescent Brain Cognitive Development (ABCD) Study®^23^ (*n* = 6437, ages 9–10 years). We hypothesised that sex differences would be greatest in association networks, as the functional topography of these networks showed the strongest associations with sex assigned at birth and cortical X-linked gene expression patterns in our previous work.^15^ Of note, the novel participant sample used in the present study differs from that used in previous work^15^ across a number of dimensions, including sample size, age range, pubertal stage, scanner types and protocols, data collection sites, functional MRI tasks, racial/ethnic diversity and socioeconomic status, allowing us to rigorously test the reproducibility and generalisability of our findings.

## Method

### Participants

Participants from the ABCD Study®^23^ baseline assessment were drawn from the ABCD BIDS Community Collection (ABCC, ABCD-3165^24^). These data were collected across 21 sites in the USA, with Institutional Review Board (IRB) approval from the University of California, San Diego, as well as from each of the respective study sites. Written informed consent (parents or guardians) and assent (children) were obtained. Criteria for participation in the ABCD Study® are described in detail in previous work.^25^ From the full baseline sample (*n* = 11 878, 9–10 years old), we excluded participants with incomplete data or excessive head motion during functional magnetic resonance imaging (fMRI) scanning (Supplementary Fig. 1, available at https://doi.org/10.1192/bjp.2025.135), yielding a final sample of *n* = 7459. Analyses were conducted in matched discovery (*n* = 3240, 50.46% female) and replication (*n* = 3197, 49.13% female) samples drawn from the ABCD Reproducible Matched Samples (ARMS^24,26^), with siblings excluded separately in the discovery and replication samples to avoid leakage across subsamples during model cross-validation (Supplementary Fig. 1). Importantly for the present study, we note that participant ‘sex’ was assessed using a binary caregiver-reported question regarding the assignment of sex at birth on the original birth certificate. Hereafter, we use the term ‘sex’ to refer to sex assigned at birth, the term ‘female’ to refer to individuals assigned female at birth, and the term ‘male’ to refer to individuals assigned male at birth. Demographic information for the participants included in the present study is presented in Supplementary Table 1.

#### fMRI processing

As in our prior work,^21,27^ we leveraged data from the ABCD BIDS Community Collection (ABCC) 3165 processed with the ABCD-BIDS pipeline, which included distortion correction and alignment, Advanced Normalization Tools (ANTS^28^) denoising, FreeSurfer^29^ segmentation and surface and volume registration with rigid-body transformation.^30,31^ Following this, further processing was done using the DCAN BOLD Processing (DBP) pipeline, which includes de-meaning and de-trending of fMRI data with respect to time; denoising using a general linear model with regressors for signal and movement; bandpass filtering between 0.008 and 0.090 Hz using a second-order Butterworth filter; applying the DBP respiratory motion filter (18.582–25.726 breaths per minute); and applying DBP motion censoring (frames exceeding a framewise displacement threshold of 0.2 mm or failing to pass outlier detection at ±3 standard deviations were discarded). We then concatenated cleaned time series data for resting-state and task-based scans, as in previous work,^21,27^ to maximise the data available for analysis. We excluded participants who had fewer than 600 remaining repetition times following motion censoring, as well as those who failed ABCD quality control for their T1 or resting-state fMRI scan.

### Definition of PFNs

**Fig. 1:**
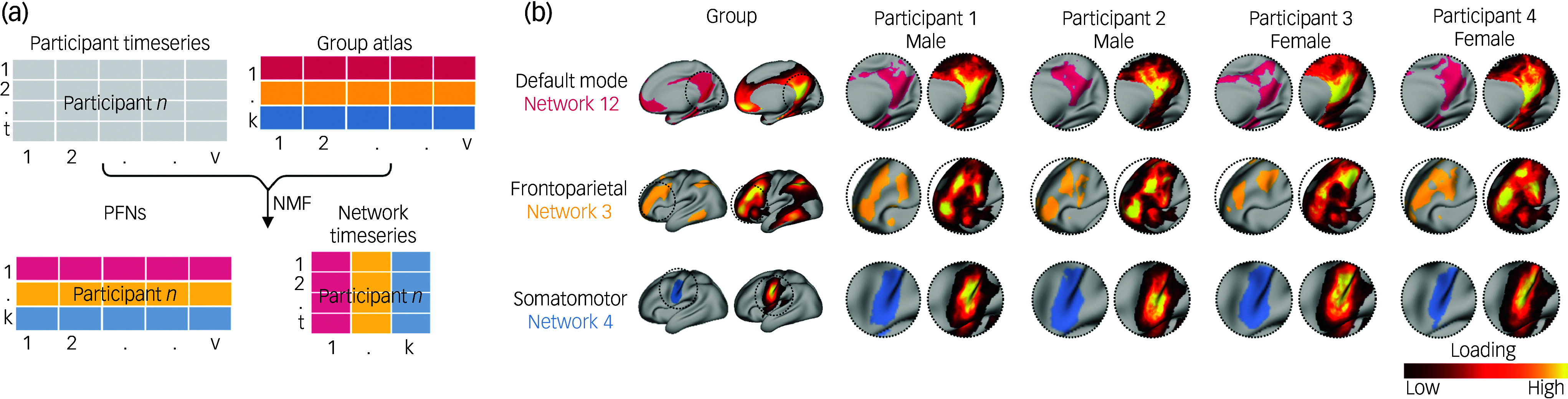
Definition of personalised functional networks (PFNs). (a) We employed a precision brain-mapping approach that leverages spatially regularised, non-negative matrix factorisation (NMF) to define individual-specific atlases of functional brain network organisation. In this approach, NMF is performed using a previously derived group consensus atlas (17 × 59 412) and each individual’s functional magnetic resonance imaging time series. This yields a 17 × 59 412 loading matrix for each participant, where each row represents a network (k), each column represents a vertex (v) and each cell represents the extent to which each vertex belongs to a given network. This probabilistic definition can be converted into discrete network definitions for display by labelling each vertex according to its highest loading. This procedure also yields a network timeseries matrix representing blood oxygen level dependent activity at each timepoint (t) for each network (k). (b) Probabilistic and discrete parcellations of three networks are displayed for the group average and four randomly selected participants. PFNs capture distinct inter-individual differences in topographic features. Inter-individual variation in topographic features is particularly prominent in association networks such as the default mode network and frontoparietal network. In contrast, sensory and motor networks are more consistent across individuals.

Detailed information about the neuroimaging acquisition for the ABCD Study®, including scanner manufacturers and MRI scanning protocols, has been described previously.^32^ Following the same fMRI preprocessing steps (Supplementary Information) as in our prior work in this data-set,^21,27^ we maximised the available high-quality data for our analyses by concatenating fMRI time series from three task-based scans (Emotional N-Back Task, Stop-Signal Task and Monetary Incentive Delay Task) and two resting-state scans, and retained only those individuals passing strict motion correction (a minimum of 600 remaining repetition time in total following motion censoring). Functional brain regions comprising large-scale networks have been shown to vary substantially in their size, shape and spatial location across individuals.^16,17^ We therefore employed a precision brain-mapping approach, as in previous work,^3,15,19,21,27^ that leverages spatially regularised, non-negative matrix factorisation (NMF)^33^ to define individual-specific atlases of functional brain network organisation (Fig. 1(a)).^3,34^ This approach has been implemented in previous studies using this data-set^21,27^ to identify 17 PFNs, revealing substantial inter-individual differences in the spatial layout of functional brain regions, with the greatest heterogeneity in association networks (Fig. 1(b)).

### Statistical analyses

We aimed to (a) evaluate whether individual-specific patterns of PFN topography were associated with sex and (b) assess the extent to which sex can be accurately classified from patterns of PFN topography in new individuals. To this end, we first conducted a mass univariate analysis relating vertex-wise PFN topography to sex, then trained multivariate classification models using rigorous cross-validation, as described in detail below.

#### Mass univariate analysis

To determine whether sex is associated with distinct patterns of PFN topography, we first evaluated vertex-wise associations, as in our previous work,^15^ using generalised additive models (GAMs) with penalised splines. These GAMs were fit at each vertex and included a linear covariate for in-scanner head motion (mean fractional displacement), a non-linear term for age and a random effect covariate for data collection site. We accounted for multiple comparisons within each PFN by controlling the false discovery rate (FDR; *Q* < 0.05). Spatial maps of GAM loadings were compared across discovery and replication samples using conservative spin-based permutation testing to account for spatial autocorrelation.^35^ To determine the role of pubertal development and hormone levels in shaping potential sex differences in PFN topography, we also conducted mass univariate analyses using data from the Pubertal Development Scale (PDS)^36^ and salivary hormone levels for dehydroepiandrosterone (DHEA), testosterone and oestradiol.^37^ DHEA and testosterone were collected for both sexes; oestradiol was collected for females only.

#### Multivariate classification

To leverage the high-dimensional data from individual-specific patterns of PFN topography across the whole cortex simultaneously, we next trained a linear support vector machine (SVM) to categorise participant sex based on their multivariate PFN loadings matrix. SVM is a common form of classifier that is well suited to leveraging high-dimensional data for binary classification, and has been shown to perform well in previous work.^15^ Replicating the procedure in our prior work,^15^ we applied nested, twofold cross-validation (2F-CV), with the inner loop used to determine the optimal tuning parameter *C* to balance model bias and variance, and the outer loop used to estimate model accuracy in held-out data. Classifier performance was evaluated using accuracy, sensitivity, specificity and the area under the receiver operating characteristic (ROC) curve. We also evaluated classifier performance relative to a set of 1000 null models, where participant sex was permuted relative to PFN topography on each iteration.

Prior to model training and testing, we eliminated siblings to avoid leakage of family structure across subsamples, yielding a total sample of *n* = 6437 (discovery: *n* = 3240, 50.46% female; replication: *n* = 3197, 49.13% female) for all multivariate classification analyses. Before beginning our 2F-CV procedure, we first split the data between the matched discovery and replication samples according to the previously defined ABCD Reproducible Matched Samples.^24,26^ Then, separately within the discovery and replication samples, we performed 2F-CV as follows (Supplementary Fig. 2). For the outer 2F-CV loop, we trained and tested the SVM model using split-half subsets separately within either the discovery or replication sample. After training the model in one half of the data and testing its performance in the other held-out half, we then repeated this procedure in reverse. Prior to model training, covariates for age, site and in-scanner head motion (mean framewise displacement) were regressed from each feature, separately in the training and testing sets to avoid leakage. To determine whether classification accuracy was driven by the choice of split, we repeated this analysis using 100 permuted splits of the data, each time randomly dividing the discovery and replication samples into independent training and testing sets.

Inner 2F-CV loops were used to determine the optimal tuning parameter *C* by further randomly dividing the training set of the outer 2F-CV loop into two subsamples. The first split-half subsample was used to train the SVM model with each of 15 possible *C* parameter values: [2^−5^, 2^−4^, …, 2^8^, 2^9^]. These models were each tested in the second held-out subsample as in our previous work.^15^ We then repeated this procedure using the second held-out subsample for training and the first subsample for testing, calculating the average held-out classification accuracy across the two subsamples for each value of the parameter *C*. The optimal *C* parameter value was selected as the *C* with the highest average held-out classification accuracy, and this optimal *C* parameter was used to train the models within the outer 2F-CV loop. It is worth noting that even the smallest subdivisions of the data in our nested 2F-CV procedure still contained >1000 participants each at a minimum, yielding sufficient statistical power to train and test our machine learning models using the most conservative possible (fewest folds) cross-validation approach.

To evaluate the relative importance of each feature within the SVM model, we first extracted feature weights for each network loading at each vertex and averaged these weights across the 100 randomly permuted splits of the data. Then, to avoid challenges with interpretation due to the covariance structure among feature weights, we applied Haufe transformation^38^ to invert the models prior to feature weight interpretation. Next, we averaged the Haufe-transformed weight maps across the training and testing sets from the outer loop of the matched-samples 2F-CV procedure. As in our univariate analysis, spatial maps of SVM weights were compared across samples using spin-based permutation testing.^35^

## Results

### Association between sex and person-specific functional topography

To characterise sex differences in functional brain network topography just prior to the transition from childhood to adolescence, we leveraged previously defined maps of PFNs (Fig. 1) for each individual in the ABCD Study® data-set^21^ (*n* = 6437, 9–10 years old, 49.8% female). These maps reflect each individual’s unique functional topography of 17 canonical large-scale networks. To determine whether a participant’s sex is reflected in their person-specific patterns of functional brain network organisation, we first conducted mass univariate analyses using GAMs to relate vertex-wise PFN loadings to sex.

**Fig. 2:**
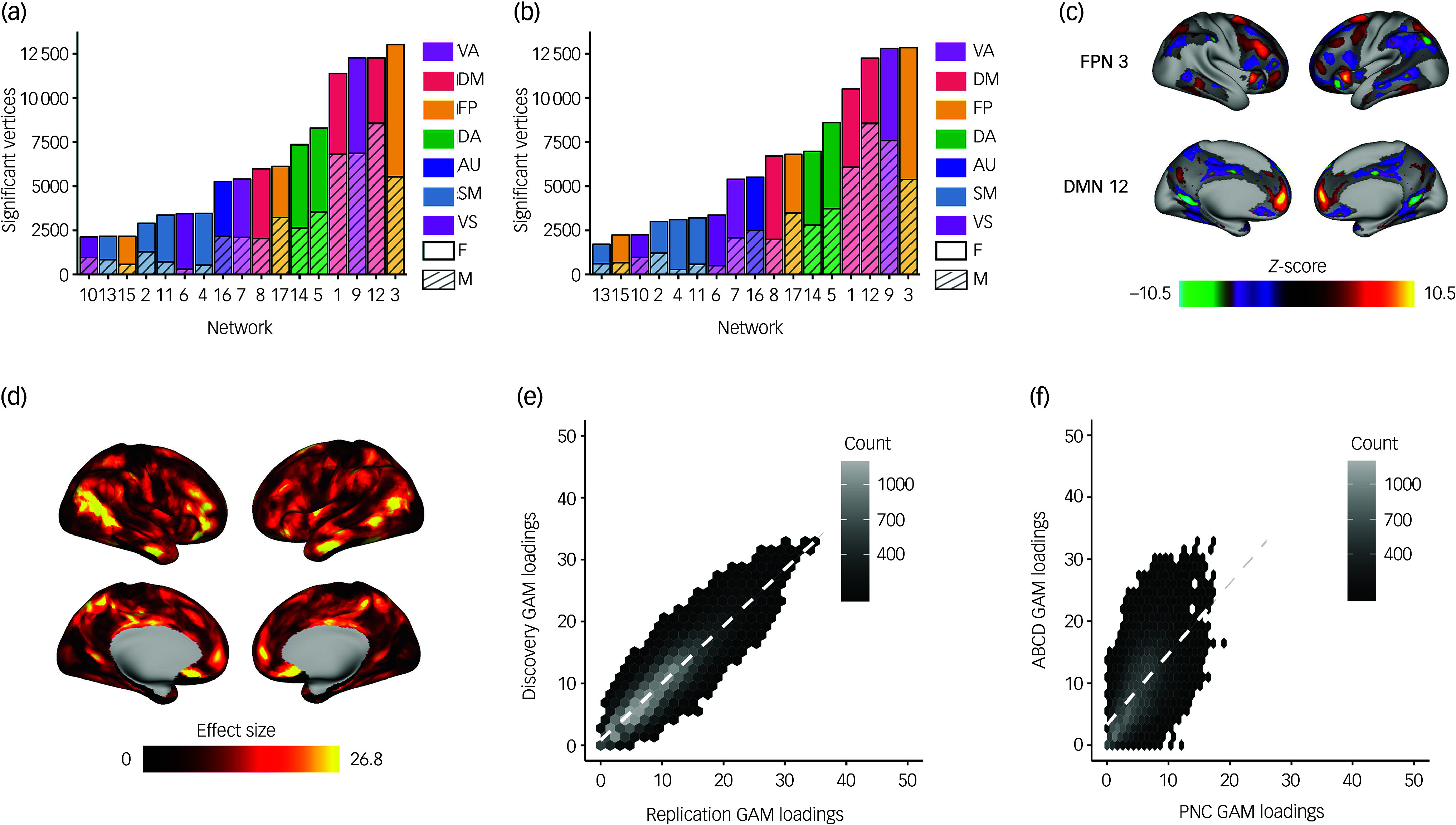
Univariate analysis identifies that sex differences are greatest in association networks. We fit a generalised additive model (GAM) at each vertex to determine the impact of sex on network loadings. Site, age and motion were included as covariates, with age modelled using a penalised spline and site modelled as a random effect. We accounted for multiple comparisons within each network with false discovery rate (*Q* < 0.05). (a) The number of vertices in each network with significant sex effects was summed separately for males and females within the discovery set. This process revealed that sex differences were greatest in the association cortex, specifically the frontoparietal, default mode and ventral attention networks. (b) The same analysis was conducted within the replication set, which yielded convergent results identifying the same three networks as having the greatest sex differences. (c) Significant vertices are displayed for the frontoparietal and default mode networks from the discovery set, as these networks were among those with the greatest sex differences. (d) The absolute sex effect across 17 networks was summed to examine the overall effect of sex at a given vertex. The summary measure depicted from the discovery set shows that the areas with the greatest sex effects are in association cortices. (e) The hexplot shows agreement between discovery and replication samples in the association between sex and network loadings (*r* = 0.90, *P*_spin_ < 0.001). (f) This hexplot shows agreement between the discovery sample in the ABCD Study^®^ and an independent data-set (Philadelphia Neurodevelopmental Cohort, PNC) from our previous report^15^ (*r* = 0.59, *P*_spin_ < 0.001) in the associations between sex and network loadings. FP/FPN, frontoparietal network; VA, ventral attention; DA, dorsal attention; DM/DMN, default mode network; AU, auditory; SM, somatomotor; VS, visual; F, female; M, male.

We found spatially heterogeneous associations between sex and PFN topography in both discovery and replication samples. Sex differences in functional topography were greatest in association networks (Fig. 2(a)–(c) and Supplementary Figs. 3 and 4), with some PFNs exhibiting greater loadings in females (e.g. frontoparietal and dorsal attention networks) and others exhibiting greater loadings in males (e.g. default mode and ventral attention networks). We evaluated the total effect of sex at each vertex by summing the absolute value of the *z*-statistic across all 17 PFNs. This analysis revealed that associations between sex and PFN topography are greatest in association cortices such as the inferior parietal lobule, ventrolateral prefrontal cortex and orbitofrontal cortex (Fig. 2(d) and Supplementary Fig. 5). We observed highly consistent spatial distributions of GAM loadings across discovery and replication samples (*r* = 0.90, *P*_spin_ < 0.001; Fig. 2(e)) and with our prior work in an independent data-set^15^ (*r* = 0.59, *P*_spin_ < 0.001; Fig. 2(f)), using conservative spin-based spatial randomisation testing to account for spatial autocorrelation.^35^ These results were also found to be consistent in sensitivity analyses that included pubertal stage, pubertal timing and salivary hormone levels as covariates (Supplementary Figs 6–9), and we observed no significant associations between PFN topography and pubertal measures, including pubertal stage, pubertal timing and salivary hormone levels (Supplementary Figs 10 and 11).

**Fig. 3:**
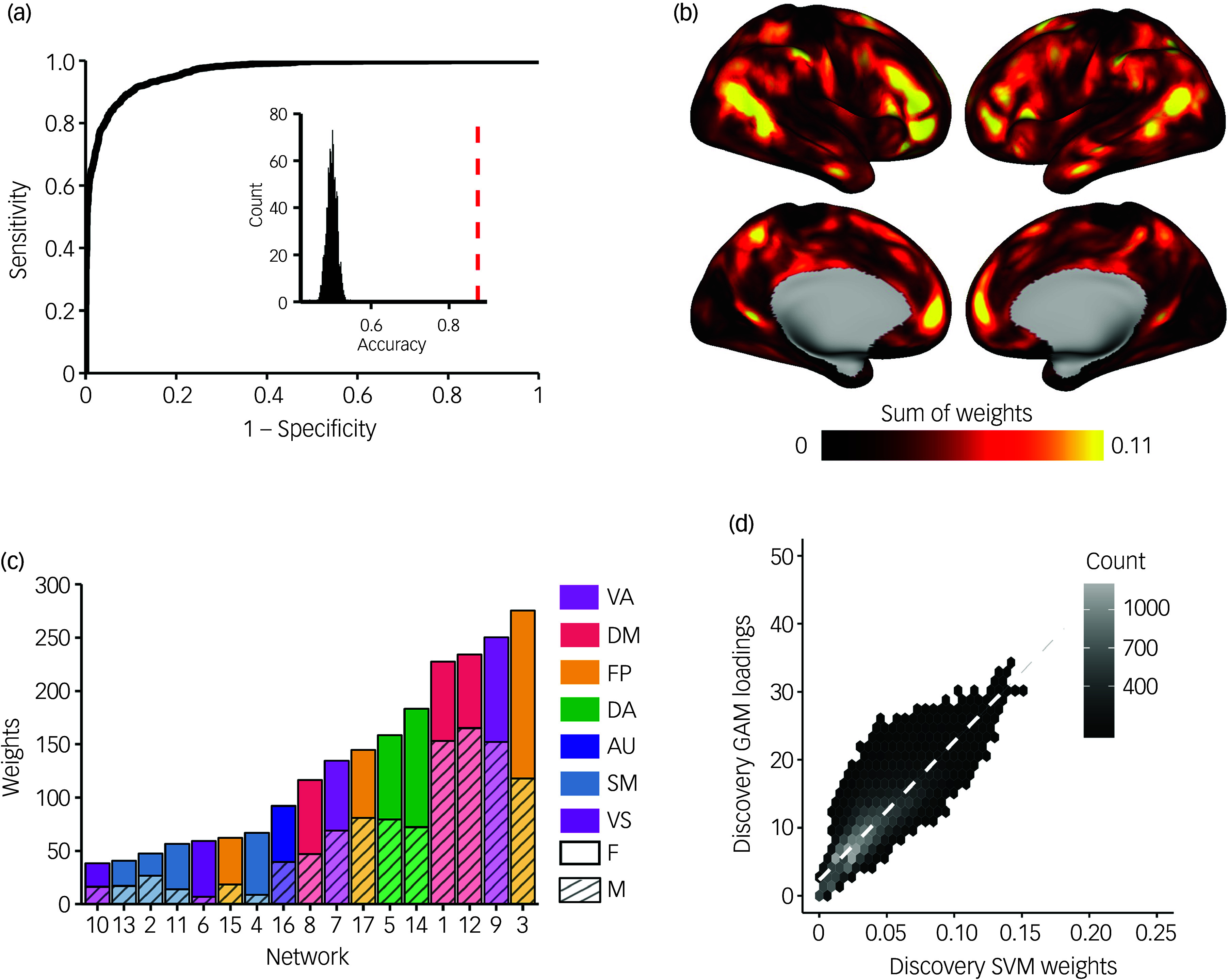
Support vector machine (SVM) models classify participant sex based on personalised functional network (PFN) topography. SVMs were trained with nested, twofold cross-validation (2F-CV) to classify participants’ sex (male or female) from PFN functional topography. (a) Depiction of the average receiver operating characteristic (ROC) curve from 100 SVM models with permuted split-half, train-test participant assignments. Average area under the ROC curve was 0.96; average sensitivity and specificity were 0.88 and 0.87, respectively. Inset histogram shows the null distribution of classification accuracies where participant sex was randomised, with the average accuracy from true (non-randomised) data represented by the dashed red line. (b) The absolute values of the feature weights were summed at each location across the cortex, revealing that association cortices contributed most to the classification of sex. (c) Positive and negative feature weights were summed separately across all vertices in each network to identify which networks contributed most to the classification. Association networks, namely the frontoparietal, ventral attention and default mode networks, were identified as the most important contributors to the classification. (d) Hexplot showing agreement between the absolute summed weights from the multivariate SVM analysis and loadings from the mass univariate generalised additive model (GAM) analysis in the discovery sample (*r* = 0.85, *P*_spin_ < 0.001). All panels represent results from the discovery sample. See Supplementary Fig. 12 for results from the replication sample and Supplementary Fig. 13 for comparison of SVM weights between the discovery and replication samples. FP, frontoparietal; VA, ventral attention; DA, dorsal attention; DM, default mode; AU, auditory; SM, somatomotor; VS, visual; F, female; M, male.

Next, we sought to confirm these vertex-wise mass univariate results by using multivariate classification to leverage the full pattern of PFN topography across the cortex. To evaluate how multidimensional patterns of PFN topography relate to participant sex, we trained linear SVM classifiers to categorise participants’ sex from PFN topography patterns using conservative cross-validation. These models were able to correctly identify held-out participants’ sex as either male or female from PFN topography patterns with high accuracy averaged across the 100 SVM iterations within each subsample (discovery, 87.4%; replication, 87.2%; Fig. 3(a) and Supplementary Fig. 12(a)), successfully replicating our prior work.^15^ Model sensitivity and specificity were 0.876 and 0.872, respectively, in the discovery sample (replication: 0.870 and 0.870), with a large area under the ROC curve (discovery, 0.966; replication, 0.965), indicating excellent model performance on held-out data that exceeded chance-level accuracy from randomly permuted null models (mean, 0.50; *P* < 0.001; Fig. 3(a) and Supplementary Fig. 12(a), inset histograms).

Model performance was robust to the choice of split in participants between the training and testing sets, as evidenced by repeated random cross-validation (discovery: mean accuracy 87.4%, 95% CI [0.873, 0.875]; replication: mean accuracy 87.2%, 95% CI [0.871, 0.873]). To identify which brain regions contributed most to the correct classification of participant sex from functional topography, we examined the SVM feature weights after applying Haufe transformation^38^ to invert the models for interpretability. Replicating prior results,^15^ we found that association networks contributed most to the classification of participant sex, primarily those within the frontoparietal, ventral attention and default mode networks (Fig. 3(b), (c) and Supplementary Fig. 12(b), (c)). Vertex-wise patterns of feature weights also provided convergent results with mass univariate analyses (discovery: *r* = 0.86, *P*_spin_ < 0.001; replication: *r* = 0.83, *P*_spin_ < 0.001; Fig. 3(d) and Supplementary Fig. 12(d)). The spatial pattern of feature weights was also highly consistent across samples (*r* = 0.93, *P*_spin_ < 0.001; Supplementary Fig. 13). We also found convergent results when SVM models were trained separately on vertex-wise loadings from each PFN independently, with ventral attention, default mode and frontoparietal networks showing the best model performance across discovery and replication samples (Supplementary Fig. 14).

## Discussion

Our results demonstrate robust and replicable sex differences in the spatial patterning of functional brain networks in youth. Across analytic approaches and independent samples, we consistently find that the spatial patterning of person-specific functional brain networks significantly differs based on sex as a biological variable. While no single brain region or network is systematically larger or smaller in its spatial extent across all males or females, we find that the greatest sex differences in functional topography tend to be disproportionately found in association areas such as the frontoparietal, default mode and ventral attention networks, with weaker effects found in sensory and motor cortices. These results represent a successful replication of prior findings^15^ in a large sample of participants, and suggest that sex might be one of many factors that shape the development of functional networks in youth at the precipice of the critical transition to adolescence. By characterising sex differences in functional topography in youth, this study provides a key stepping stone towards addressing sex differences in susceptibility to psychiatric symptoms that emerge during the transition to adolescence.

### Sex differences in personalised functional brain network topography in youth

Extending prior work describing sex differences in neuroimaging features in young adults,^39,40^ our results suggest that sex differences in functional topography are consistently observed in children just prior to the transition to adolescence. This critical transition period that often coincides with pubertal changes is marked by the emergence of many common psychiatric disorders, including depression and anxiety, which disproportionately affect females.^1^ This time period also coincides with the maturation of functional brain networks, including the protracted development of association networks such as the frontoparietal and default mode networks,^41,42^ which have been shown to have distinct profiles of functional development between males and females.^43^ These association networks also exhibit the most person-specific patterns of functional topography among all large-scale brain networks,^3^ and are associated with symptoms of psychopathology.^19,20^ Our observation that these networks also reflect an individual’s sex aligns with previous findings,^44^ including recent findings in adults,^39,40^ and suggests that sex differences in functional brain networks may play a role in the emergence or exacerbation of sex differences in psychiatric disorders during the transition to adolescence. Thus, future studies may seek to further investigate potential behavioural consequences of sex differences in association network topography in youth, as well as the potential role of functional brain network development as an early biomarker for sex-specific psychiatric symptom emergence in youth.

### Sex differences in functional topography consistently replicate across independent data-sets

Replication studies often fail,^45^ and even successful replication studies most often yield results with smaller effect sizes than initial discoveries.^46^ The present study not only successfully replicates findings observed in our prior work, but also uncovered effect sizes that were approximately the same or even larger than in the previous study.^15^ Specifically, the present study confirmed the presence of sex differences in PFN topography and replicated the observation that these differences are primarily found in association networks. This successful replication is especially notable in light of the many differences between the data-sets in each study, including sample size, age range, scanner types and protocols, data collection sites, fMRI tasks, racial/ethnic diversity and socioeconomic status. Thus, the present study represents a strong counterexample to the ongoing reproducibility crisis in psychology and neuroscience.^22^

Several important distinctions between the present study and this previous work provide context for interpreting these results. First, the previous study^15^ used data from the Philadelphia Neurodevelopmental Cohort (PNC; *n* = 693); here, we applied the same analytical approach to a data-set that is an order of magnitude larger (ABCD Study®; *n* = 6437). This considerable increase in sample size may explain the improvement in model performance on held-out data between studies (from 82.9 to 87.1% accuracy), as models trained in larger data-sets with rigorous cross-validation are less likely to be overfit.^47,48^ Second, the previous study^15^ assessed individuals aged 8–23 years old while the present study leveraged data from the baseline assessment of the ABCD Study® when participants were 9–10 years old. The more restricted age range in the present study may also help to explain the improved model performance, since functional brain network topography changes throughout development.^2,3^ Although age was included as a model covariate in both studies, it is possible that the smaller age range in the present study still yielded some advantage in classifying sex from patterns of functional topography at a more restricted time period of brain development.

### Limitations

There are several limitations of this study worth noting. First, sex was assessed using a binary parent-reported question regarding the assignment of sex at birth on the original birth certificate, and we lacked a sufficiently large sample size to examine functional topography of intersex youth. Importantly, existing data suggest that binary classifications of sex do not align well with the complex mosaics of male and female characteristics observed in individual brains.^49^ Thus, further research is warranted to more comprehensively characterise person-specific patterns of male, female and intersex characteristics in functional brain network topography. Second, prior work has shown that functional brain network connectivity is associated with both sex and gender in youth.^50^ Because the present study aimed to understand sex differences in functional topography, future work is also needed to investigate the potential effects of continuous gender dimensions such as gender identity and expression. Given that only 0.5% (*n* = 58) of baseline ABCD Study® participants reported being, or possibly being, transgender,^51^ and given that gender continues to develop throughout early adolescence, future studies in longitudinal timepoints will be key in investigating potential individual or interactive effects of sex and gender in shaping neurodevelopment.

Third, the present study leveraged a cross-sectional sample at a single time point from within an ongoing longitudinal study of youth. As youth from the ABCD Study® continue to participate in follow-up study sessions from childhood to adulthood, it will become increasingly possible to investigate changes in sex-specific functional brain network topography with critical developmental changes such as puberty across longer time scales than investigated in the present study. Moreover, because puberty was already under way in a substantial portion of females in the ABCD Study®, future studies of younger individuals will be required to investigate the activational role of pubertal hormones, which begin before physical changes become observable, on sex differences in functional topography. Future longitudinal studies considering the complex interplay of biopsychosocial factors related to sex and gender development may also reveal mechanistic links between sex-specific patterns of functional brain network topography and sex differences in psychiatric illness manifestation (e.g. internalising symptoms). Fourth, the present study focused on sex differences in functional rather than structural differences in brain organisation, although sex differences in gross structural anatomy (e.g. head size) are well documented.^52^ However, recent work has demonstrated that sex differences in functional brain organisation do not appear to be systematically associated with structural imaging measures such as surface area or microstructural organisation.^44^

### Future directions: using precision brain mapping to inform female mental health

In addition to the future directions noted above, our observation that person-specific patterns of functional brain network topography show sex differences, particularly in association networks related to psychiatric symptoms,^19,20^ also lays important groundwork for future studies of sex differences in mental health, including mental health conditions that disproportionately impact females. First, future work should further examine how PFN topography develops across the female reproductive lifespan, with a particular focus on changes across critical hormonal transition periods such as puberty, pregnancy and menopause. These hormonal transition periods are known to have substantial impact on neurodevelopment and often align with the timing of psychiatric illness onset,^53^ yet have been historically underfunded and understudied.^54^ Extending the study of PFNs across the lifespan therefore has potential to improve our understanding of how neuroplasticity during hormonal shifts impacts functional topography and trajectories of psychiatric illness. Second, longitudinal studies examining how sex differences in PFN topography emerge during development may inform early preventions or personalised treatments for psychiatric illnesses such as personalised neuromodulation via transcranial magnetic stimulation (TMS), filling critical gaps in existing treatment options.

Finally, it is worth noting that sex-specific individual differences in the topography of association networks may also reflect childhood environments and socioeconomic status,^27^ which have also been shown to explain a large portion of inter-individual variance in psychopathology symptoms.^55^ Taken together with evidence of sex differences in stress responses across the lifespan,^56^ our findings motivate future research into whether sex differences in the effects of environmental stressors are associated with sex differences in association network topography and psychiatric illness. Additionally, environmental stressors have been shown to confer vulnerability to psychiatric symptoms during future reproductive time points characterised by significant hormonal fluctuations such as pregnancy^57^ and menopause.^58,59^ Future work may therefore seek to parse the independent and interactive effects of hormonal, genetic and environmental factors that, together, may shape individual-specific spatial patterning of functional networks across the female reproductive lifespan.

## Supporting information

Keller et al. supplementary material Keller et al. supplementary material

## Data Availability

Data used in the preparation of this article were obtained from the ABCD Study^®^ (https://abcdstudy.org), held in the National Institute of Mental Health (NIMH) Data Archive (NDA). Only researchers with an approved NDA Data Use Certification (DUC) may obtain ABCD Study^®^ data.

## Supplementary material

The supplementary material is available online at https://doi.org/10.1192/bjp.2025.135

## Analytic code availability

Analytic code used in this study is available at https://ashleychari.github.io/abcd_sex_pfn_replication/
