# Supplementary material for: Reproducible sex differences in personalised functional network topography in youth
Source: Br J Psychiatry. 2025 Jun 19;226(6):383–91. doi: 10.1192/bjp.2025.135 (PMC12245598; doi:10.1192/bjp.2025.135)
Supplement: Keller et al. supplementary material [file S0007125025001357sup001.pdf]

Supplementary Information

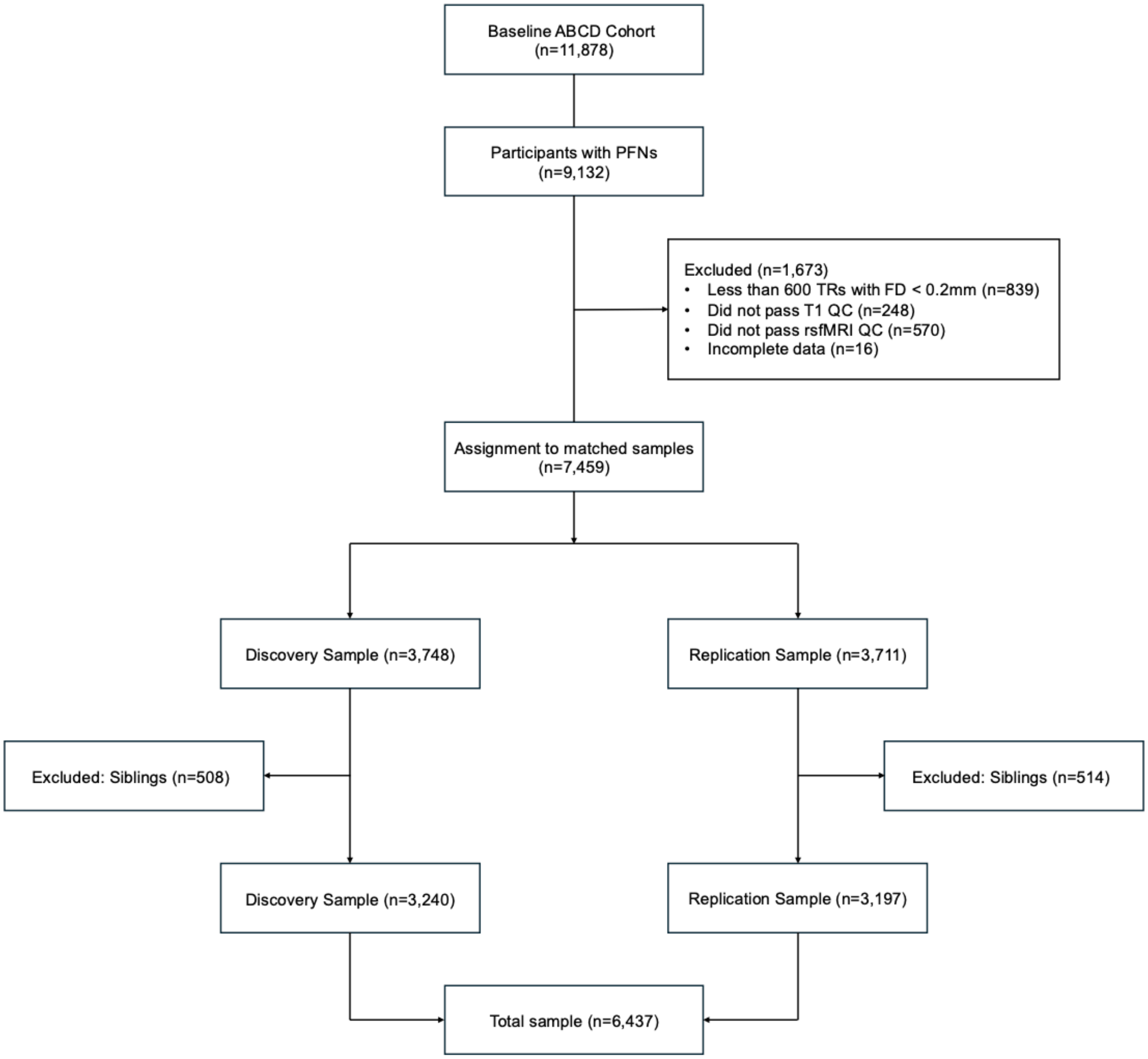

Figure S1. Flow diagram depicting data inclusion and exclusion. Participants from the Adolescent Brain Cognitive Development (ABCD) Study®[1] baseline assessment (n = 11,878) were drawn from the ABCD BIDS Community Collection (ABCC, ABCD-3165[2]). Participants were excluded for having incomplete data or excessive head motion. The matched samples were then split into discovery and replication samples according to the ABCD Reproducible Matched Samples (ARMS[2,3]). Siblings were excluded from the discovery and replication sets separately to avoid leakage across subsamples during two-fold cross-validation, yielding a total of n = 3,240 participants in the discovery sample and n = 3,197 participants in the replication sample.

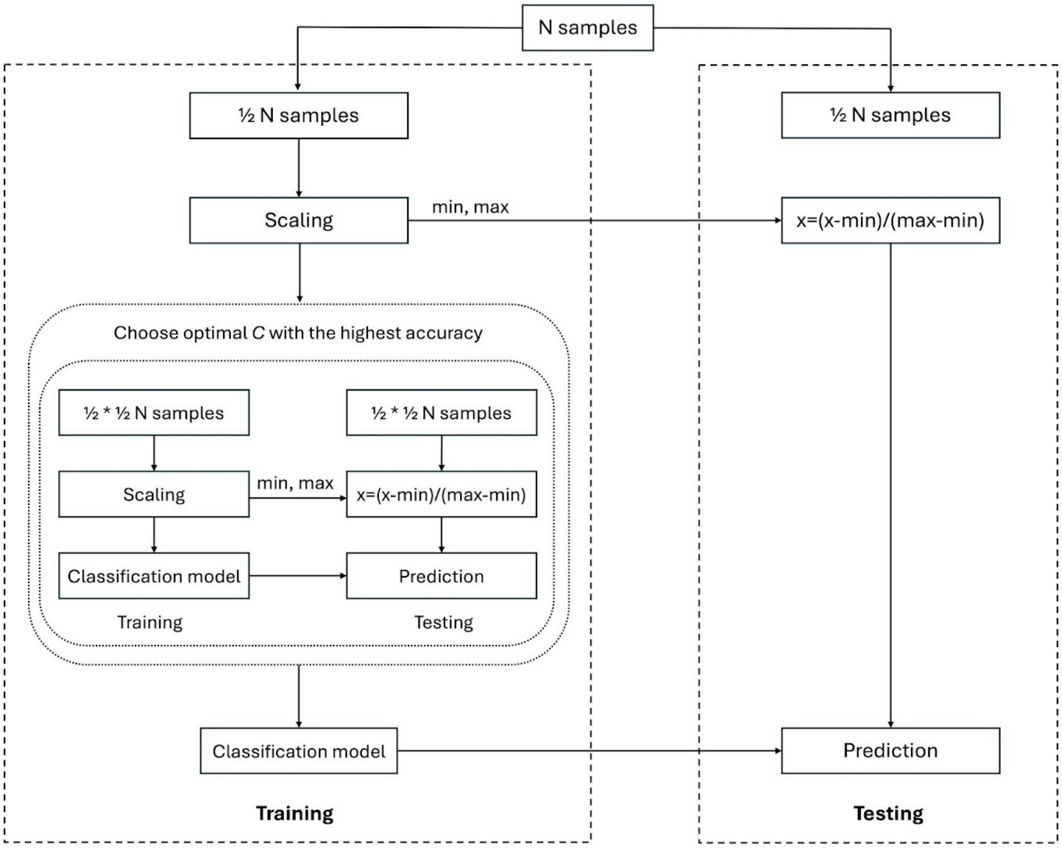

Figure S2. Schematic of Nested Two-Fold Cross Validation Procedure. Participants were first divided into matched discovery and replication samples according to the ABCD Reproducible Matched Samples (ARMS[2,3]) and siblings were removed to avoid leakage across subsamples. The discovery sample was then randomly divided into 2 subsets, with the first half used as a training set and the second half used as a testing set. Each feature was linearly scaled between zero and one across the training dataset; these scaling parameters were applied to the testing sample. An inner 2F-CV was applied within the training set to select the optimal C parameter. Based on the optimal C, we trained a model using all subjects in the training set, and then used that model to predict the outcome of all participants in the testing set. The same process was then repeated in the replication sample.

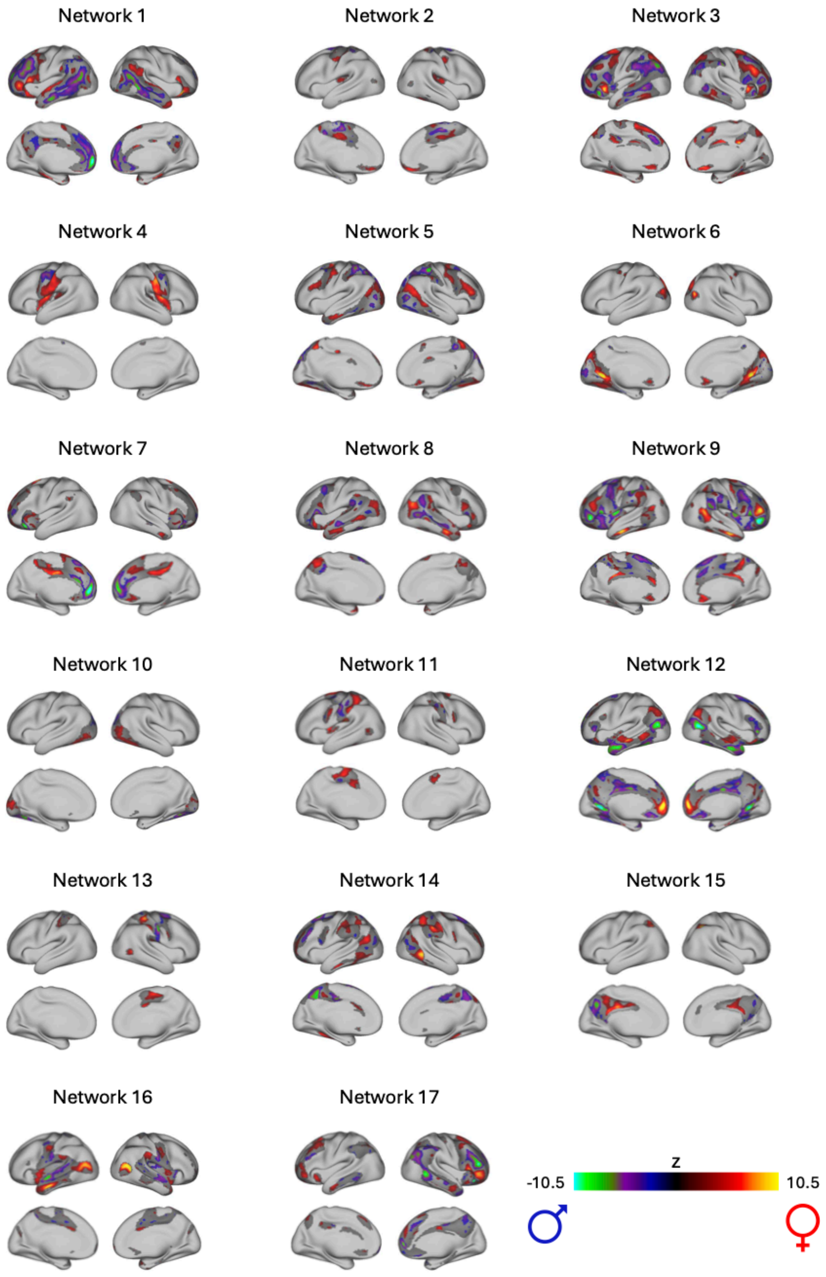

Figure S3. Univariate analyses identify significant sex differences in association networks. A GAM was fit at each vertex to evaluate the association between sex and network loadings. Age, site, and head motion were included as covariates with age modeled using a penalized spine and site modeled as a random effect. Multiple comparisons were accounted for by controlling the false discovery rate (Q<0.05). Significant vertices are shown for each of the 17 PFNs.

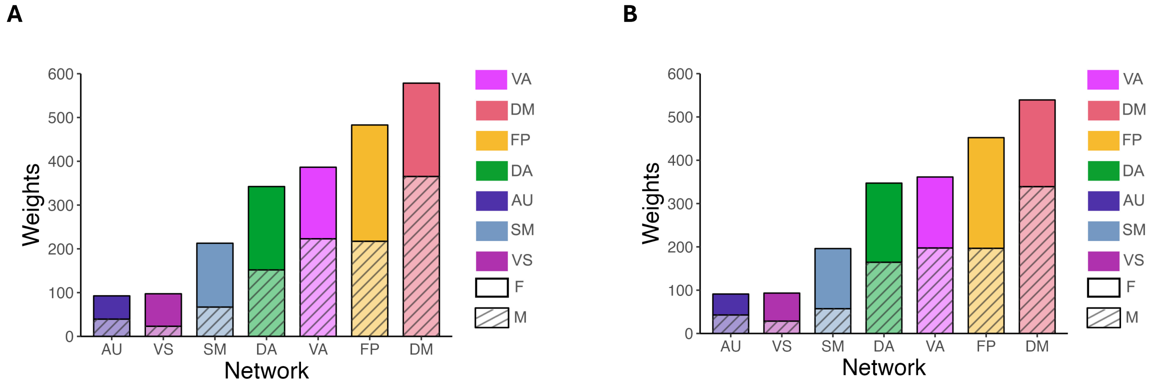

Figure S4. Univariate analysis identifies significant sex differences in association networks in both discovery dataset and replication dataset. A GAM was fit at each vertex to evaluate associations between sex and PFN network loadings. Age, site, and motion were included as covariates with age modeled using a penalized spline and site modeled as a random effect. Multiple comparisons within each network were accounted for by controlling the false discovery rate (*Q*<0.05). The number of significant vertices for each network category was summed separately for males and females (e.g., the "DM" bar represents the number of vertices with significant sex effects in networks 1, 8, and 12). Sex differences were greatest in association networks in both the discovery *(A)* and replication *(B)* samples. *Abbreviations*: FP = Fronto-Parietal; VA = Ventral Attention; DA = Dorsal Attention; DM = Default Mode; AU = Auditory; SM = Somatomotor; VS = Visual; F = Female; M = Male.

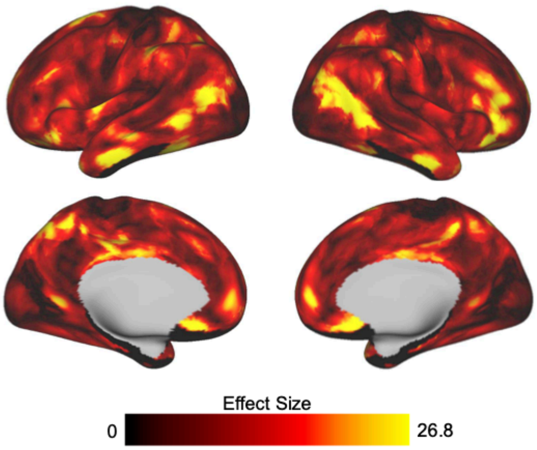

Figure S5. Univariate analysis identifies significant sex differences in association networks in the replication sample. We summed the absolute sex effect across 17 networks to examine the overall effect of sex at a given vertex within the replication sample. Brain areas with the greatest sex effects are found in association cortices.

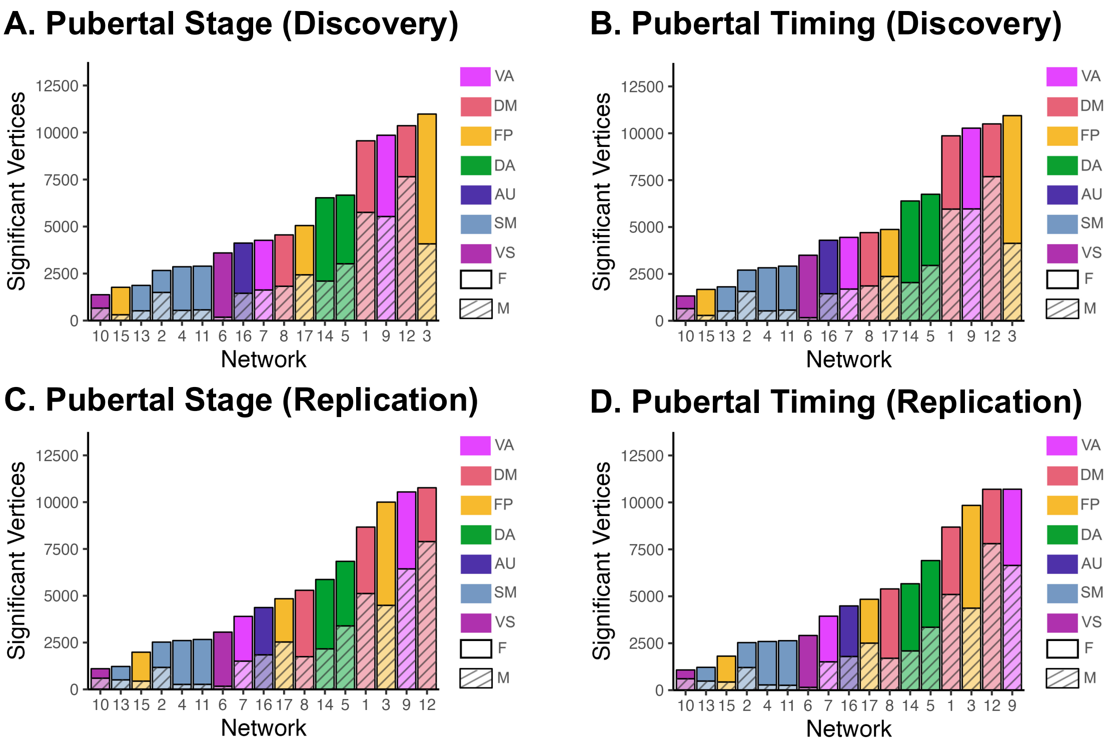

Figure S6. Sex differences in association network topography are robust to sensitivity analyses accounting for pubertal stage and timing. We conducted a sensitivity analysis using generalized additive models to identify sex differences in PFN topography while accounting for pubertal stage (Panels A, C) and pubertal timing (Panels B, D). The number of vertices in each network with significant sex effects were summed separately for males and females, revealing that sex differences were greatest in the association cortex, specifically the frontoparietal, default mode, and the ventral attention networks across the discovery (Panels A, B) and replication (Panels C, D) samples.

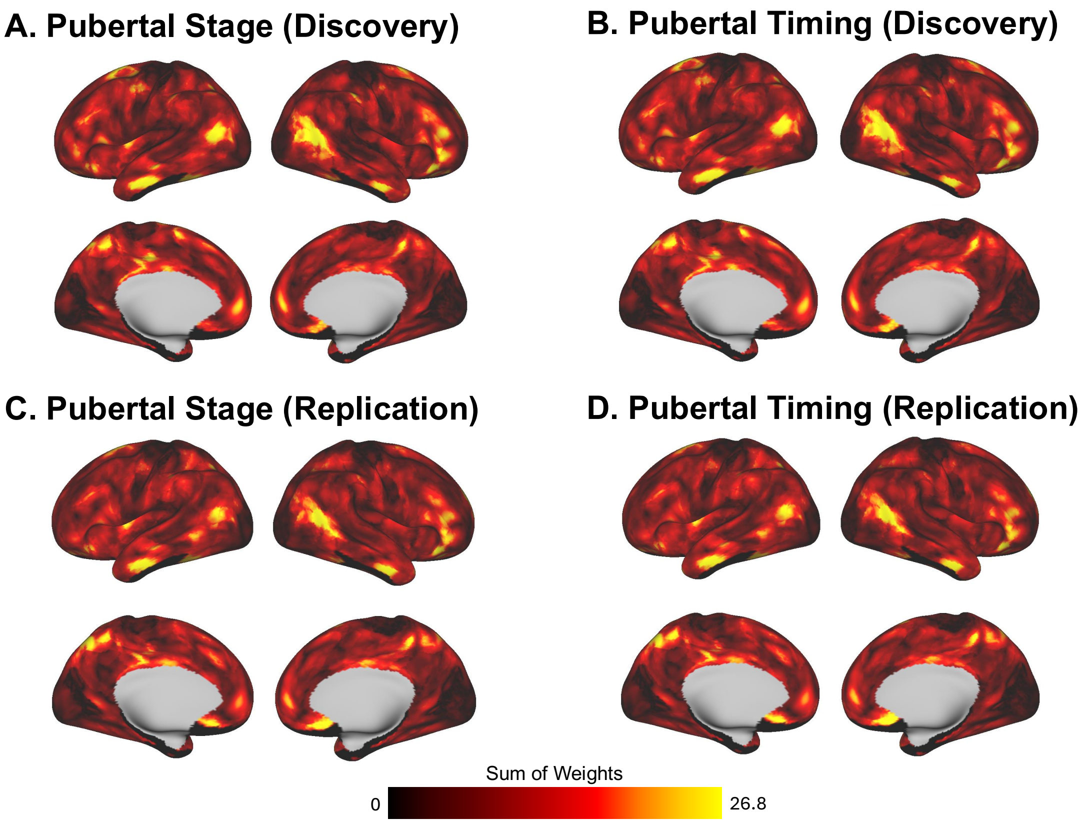

Figure S7. Vertex-wise sex differences in PFN topography accounting for pubertal stage and timing. We conducted a sensitivity analysis using generalized additive models to identify sex differences in PFN topography while accounting for pubertal stage (Panels A, C) and pubertal timing (Panels B, D). The number of vertices in each network with significant sex effects were summed separately for males and females, revealing that sex differences were greatest in the association cortex, specifically the frontoparietal, default mode, and the ventral attention networks across the discovery (Panels A, B) and replication (Panels C, D) samples.

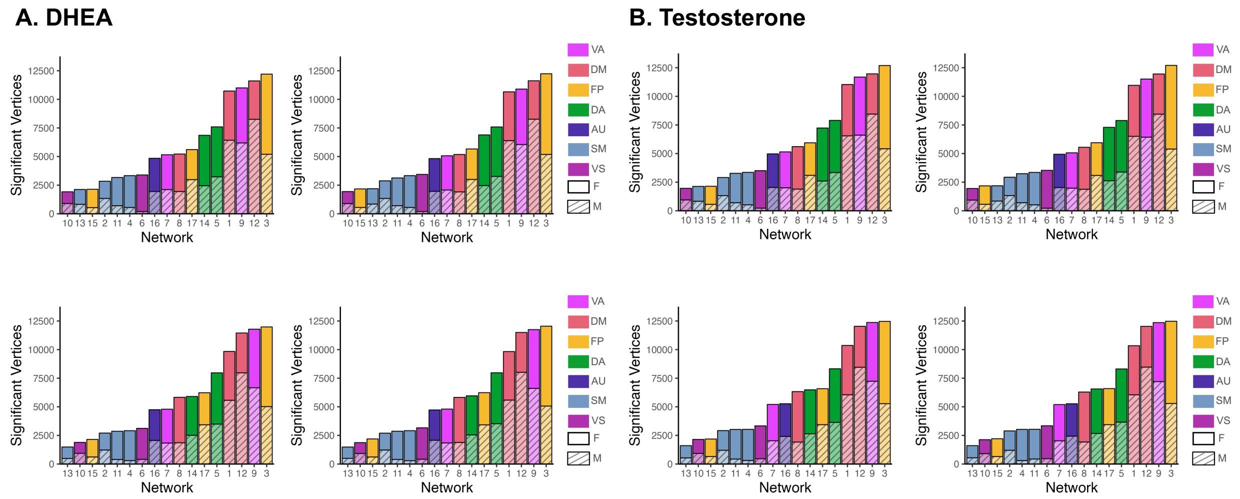

Figure S8. Sex differences in association network topography are robust to sensitivity analyses accounting for salivary hormone levels. We conducted a sensitivity analysis using generalized additive models to identify sex differences in PFN topography while accounting for salivary hormone levels in DHEA (Panel A) and testosterone (Panel B). The number of vertices in each network with significant sex effects were summed separately for males and females, revealing that sex differences were greatest in the association cortex, specifically the frontoparietal, default mode, and the ventral attention networks across the discovery (top rows) and replication (bottom rows) samples, regardless of whether age was included (left columns) or not included (right columns) in the model.

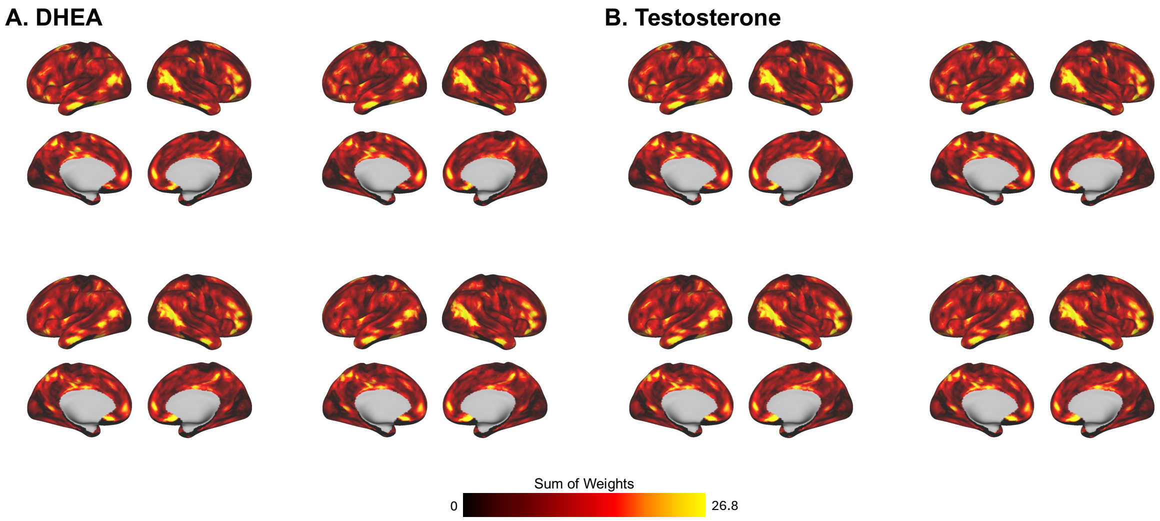

Figure S9. Vertex-wise sex differences in PFN topography accounting for salivary hormone levels. We conducted a sensitivity analysis using generalized additive models to identify sex differences in PFN topography while accounting for salivary hormone levels in DHEA (Panel A) and testosterone (Panel B). We then summed the absolute sex effect across 17 networks to examine the overall effect of sex at a given vertex, revealing that brain areas with the greatest sex effects are consistently found in association cortices across the discovery (top rows) and replication (bottom rows) samples, regardless of whether age was included (left columns) or not included (right columns) in the model.

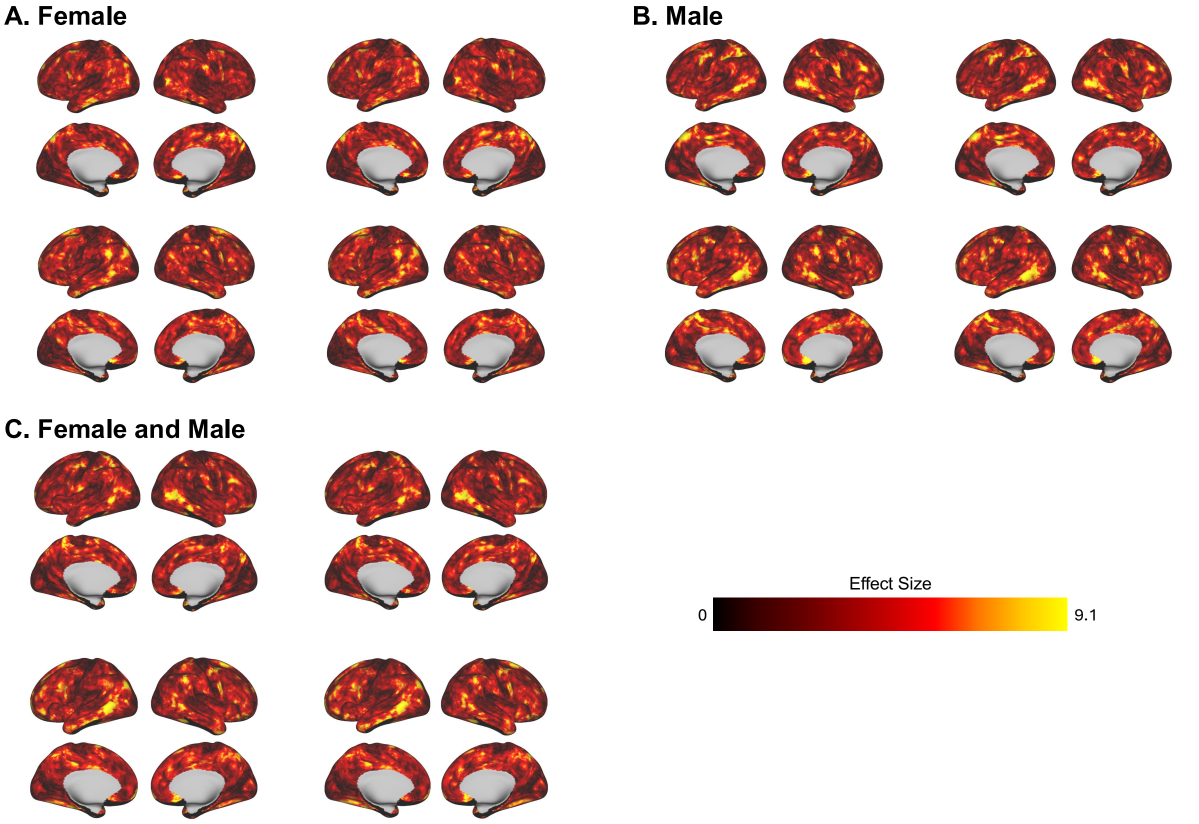

Figure S10. Associations between pubertal stage and PFN topography. The absolute effects of pubertal timing (left columns) and pubertal stage (right columns; age included as a covariate) across all seventeen PFNs were summed to examine the overall effect of puberty on PFN topography at each vertex for females (Panel A), males (Panel B), and both females and males (Panel C). Within each panel, results from the discovery sample are represented in the top row and results from the replication sample are represented in the bottom row.

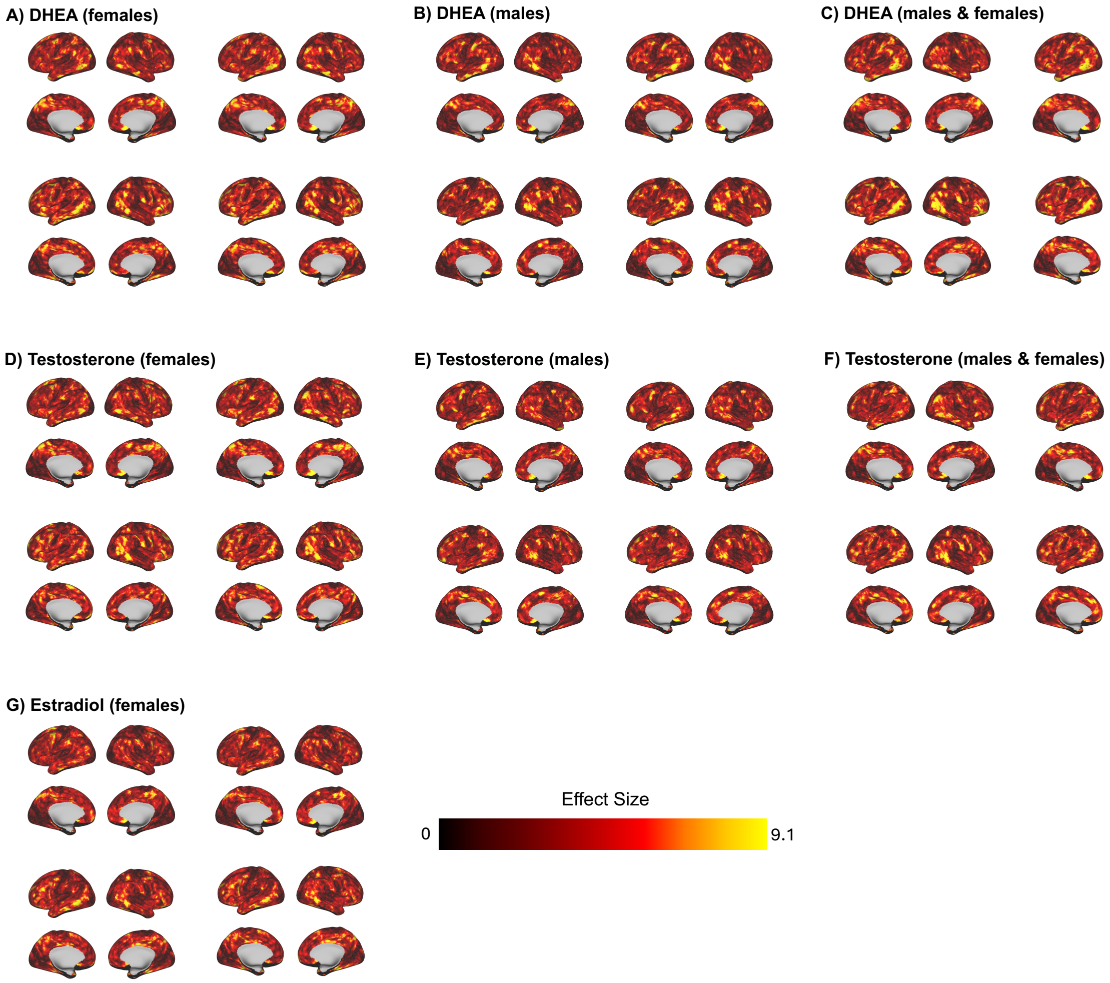

Figure S11. Associations between salivary hormone levels and PFN topography. The absolute effects of salivary hormone levels across all seventeen PFNs were summed to examine the overall effect of hormone levels at each vertex with (left columns) and without (right columns) age included as a covariate. Within each panel, results from the discovery sample are represented in the top row and results from the replication sample are represented in the bottom row.

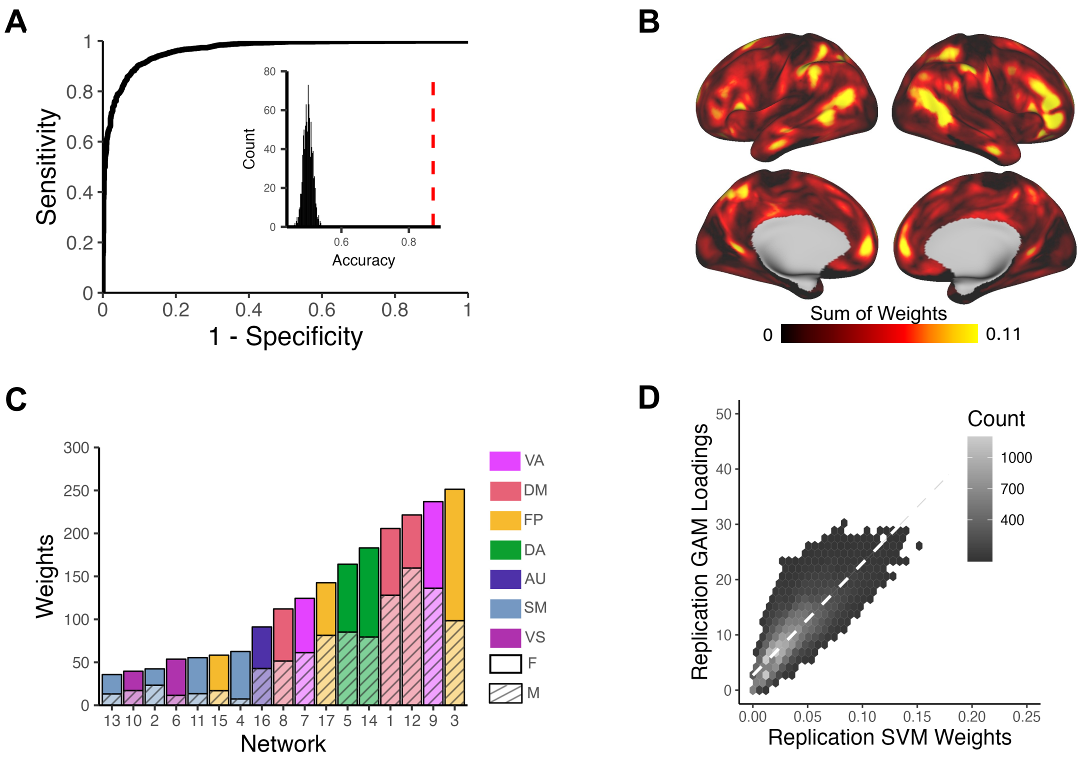

Figure S12. Support vector machines (SVMs) accurately classify participant sex from PFN functional topography in the replication dataset. SVMs were trained with nested two-fold cross-validation (2F-CV) to classify participants' sex (male or female) based on PFN functional topography. *(A)* Depiction of the average ROC curve from 100 SVM models with permuted split-half train-test participant assignments. Average area under the ROC curve was 0.96; average sensitivity and specificity were 0.87 and 0.87, respectively. Inset histogram shows the null distribution of classification accuracies where participant sex was randomized, with the average accuracy from true (non-randomized) data represented by the dashed red line. *(B)* The absolute value of the feature weights were summed at each location across the cortex, revealing that association cortices contributed most to the classification of sex. (*C)* Positive and negative feature weights were summed separately across all vertices in each network to identify which networks contributed most to the classification. Association networks, namely the fronto-parietal, ventral attention, and default mode networks, were identified as the most important contributors for classification. *(D)* Hexplot shows agreement between the absolute summed weights from the multivariate SVM analysis and loadings from the mass univariate generalized additive model analysis in the discovery sample ($r = 0.82$; $p_{spin} < 0.001$). *Abbreviations*: FP = Fronto-Parietal; VA = Ventral Attention; DA = Dorsal Attention; DM = Default Mode; AU = Auditory; SM = Somatomotor; VS = Visual; F = Female; M = Male.

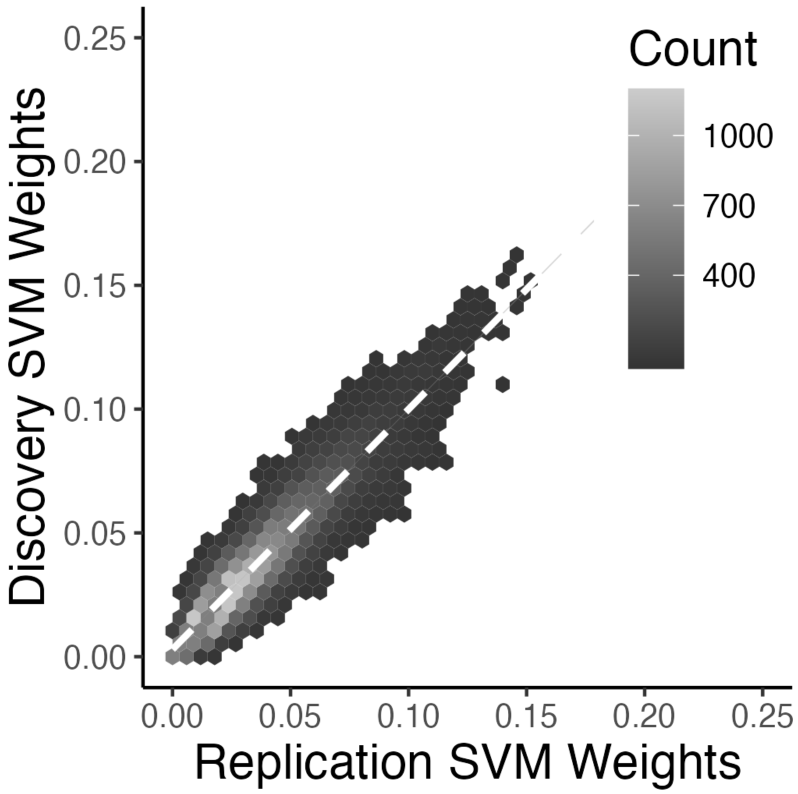

Figure S13. Comparison of support vector machine (SVM) feature weights across samples. The hexplot shows agreement between discovery and replication samples in the association between sex and network loadings from the SVM models (r = 0.93; p_{spin} < 0.001).

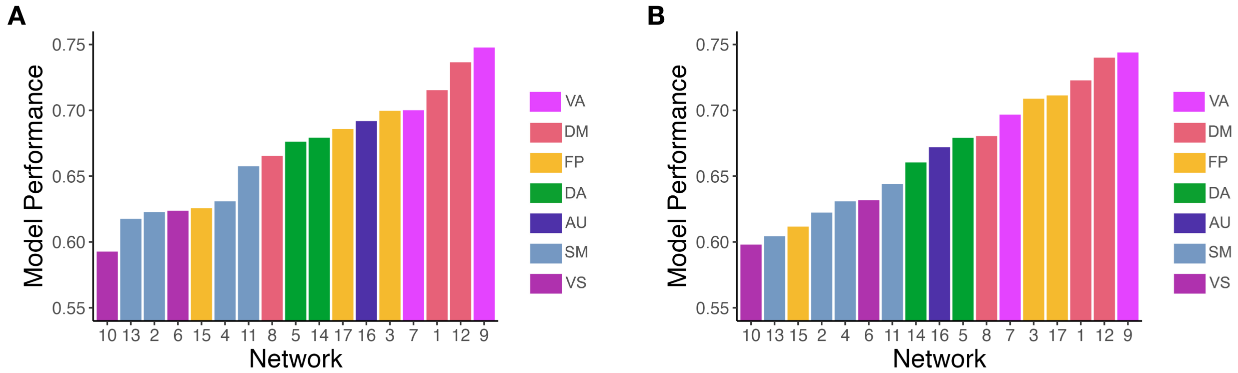

Figure S14 Support vector machine models trained on association network topography show highest classification accuracy. To determine which personalized functional networks (PFNs) showed the highest accuracy in classifying participant sex from functional topography, we trained 17 support vector machine (SVM) models on the loadings from each PFN independently in the discovery (**A**) and replication (**B**) samples. Model performance for each SVM is quantified as average classification accuracy across 100 iterations of our nested two-fold cross-validation procedure. Association networks, including the ventral attention, default mode, and fronto-parietal networks showed the highest classification accuracy across samples. Sensorimotor networks such as the visual and somatomotor networks showed lower classification accuracy. *Abbreviations*: FP = Fronto-Parietal; VA = Ventral Attention; DA = Dorsal Attention; DM = Default Mode; AU = Auditory; SM = Somatomotor; VS = Visual.

	Discovery	Replication	Total
**Age**	9.94 (0.62)	9.95 (0.62)	9.94 (0.62)
**Sex**			
M	1605 (49.54%)	1626 (50.86%)	3231 (50.19%)
F	1635 (50.46%)	1571 (49.14%)	3206 (49.81%)
**Race/Ethnicity**			
White	2194 (67.72%)	2101 (65.72%)	4295 (66.72%)
Black	426 (13.15%)	453 (14.17%)	879 (13.66%)
Asian	68 (2.1%)	73 (2.28%)	141 (2.19%)
AIAN/NHPI	22 (0.68%)	17 (0.53%)	39 (0.61%)
Mixed	371 (11.45%)	384 (12.01%)	755 (11.73%)
Other	119 (3.67%)	121 (3.78%)	240 (3.73%)
Missing	40 (1.23%)	48 (1.5%)	88 (1.37%)
**Household Income**			
[<50K]	782 (24.14%)	816 (25.52%)	1598 (24.83%)
[>=50K & <100K]	907 (27.99%)	845 (26.43%)	1752 (27.22%)
[>=100K]	1289 (39.78%)	1274 (39.85%)	2563 (39.82%)
Missing	262 (8.09%)	262 (8.2%)	524 (8.14%)
**Horomone Samples**			
DHEA	64.92 (48.93)	63.85 (48.25)	64.39 (48.59)
Testosterone	34.09 (18.2)	33.73 (18.4)	33.91 (18.3)
Estradiol	1.05 (0.49)	1.08 (0.51)	1.07 (0.5)
**PDS Female (n=3092)**			
Prepuberty	479 (30.32%)	451 (29.83%)	930 (30.08%)
Early Puberty	380 (24.05%)	368 (24.34%)	748 (24.19%)
Mid Puberty	686 (43.42%)	655 (43.32%)	1341 (43.37%)
Late Puberty	33 (2.09%)	36 (2.38%)	69 (2.23%)
Post-Puberty	2 (0.13%)	2 (0.13%)	4 (0.13%)
**PDS Male (n=3120)**			
Prepuberty	1120 (72.35%)	1101 (70.04%)	2221 (71.19%)
Early Puberty	354 (22.87%)	386 (24.55%)	740 (23.72%)
Mid Puberty	70 (4.52%)	75 (4.77%)	145 (4.65%)
Late Puberty	4 (0.26%)	10 (0.64%)	14 (0.45%)

Table S1. Demographic characteristics in matched discovery (n=3240) and matched replication (n=3197) samples. DHEA=Dehydroepiandrosterone; AIAN = American Indian/Alaska Native; NHPI = Native Hawaiian and other Pacific Islander; PDS = Pubertal Development Scale.

Supplementary References

1. Volkow ND, Koob GF, Croyle RT, Bianchi DW, Gordon JA, Koroshetz WJ, et al. The conception of the ABCD study: From substance use to a broad NIH collaboration. *Dev Cogn Neurosci* 2018; 32: 4–7.
2. Feczko E, Conan G, Marek S, Tervo-Clemmens B, Cordova M, Doyle O, et al. Adolescent Brain Cognitive Development (ABCD) Community MRI Collection and Utilities. *bioRxiv* 2021. doi:10.1101/2021.07.09.451638.
3. Cordova MM, Doyle O, Conan G, Feczko E, Earl E, Perrone A, et al. ABCD Reproducible Matched Samples (ARMS) software. *Open Science Framework* 2021.
